# A case of labio-facial necrotizing fasciitis complicating acne

**DOI:** 10.1186/s13104-016-2041-3

**Published:** 2016-04-23

**Authors:** Amel Salah Eltayeb, Abdelnasir Gafar Ahmad, Elnour Ibrahim Elbeshir

**Affiliations:** Khartoum Teaching Dental Hospital, P.O box 122, Khartoum, Sudan; Faculty of Dentistry, The University of Medical Sciences and Technology, P.O Box 12810, Khartoum, Sudan

**Keywords:** Necrotizing fasciitis, Lower lip, Angioedema

## Abstract

**Background:**

Facial necrotizing fasciitis is extremely rare. Most of the cases reported in literature are related to dental, sinus, tonsillar and salivary glands causes, but rarely as consequence of skin infection. We report a unique case of lower lip cellulitis, which was initially misdiagnosed as angioedema and subsequently progressed into lower lip necrotizing fasciitis.

**Case presentation:**

This is a case report of necrotizing fasciitis involving the lower lip as a consequence of infected skin acne in a 19 year old black female. The patient had been diagnosed earlier as a case of angioedema by a physician and treated accordingly. She was mildly anemic, hyponatremic and hypokalemic. Treatment was started immediately by incision, drainage and full debridement of the whole necrotic tissue. Triple antibiotic therapy was administered and daily irrigation and dressing were performed until full recovery. Complete healing occurred within a month by secondary intention.

**Conclusion:**

This case demonstrates the misdiagnosis of a lip swelling leading to the development of labiofacial necrotizing fasciitis, a serious and life threatening condition. Lip angioedema is a common condition; however, lip fasciitis is rare. A broad differential diagnosis in case of lower lip swelling is essential to avoid inappropriate treatment delay.

## Background

Necrotizing fasciitis (NF) is a rare but potentially fatal infection [1]. Its rapid and destructive clinical course is assumed to be caused by polymicrobial symbiosis and is usually associated with immunocompromised status such as cancer, diabetes mellitus, vascular insufficiencies, organ transplantation and alcohol abuse [2]. It most commonly presents in the extremities [leg 33 % and hand 7.5 %] [3], trunk, genitalia and perineum (20.2 %) [4]. In the head and neck region, cervical necrotizing fasciitis is rare (5.3 %) [5] but when it does occur, dental infection is a frequent cause [6]. Cervical necrotizing fasciitis is characterized by cutaneous necrosis, suppurative fasciitis, thrombosis of small blood vessels in the subcutaneous tissue, and extreme systemic toxicity. It is a severe condition with a high risk of death [7], and is aggravated by spread of the infection through the fasciae, with development of mediastinitis and septic shock. Deformity of the face and submandibular area is a frequent complication [8].

Facial NF is extremely rare. In 1997 Shindo et al. found only 35 reports of facial NF in their review [9]. The source of facial necrotizing fasciitis infection is either dental, sinus, peritonsillar or salivary gland infections. Infections secondary to surgery or trauma have also been reported as causative factors. Group A beta-hemolytic streptococci and staphylococci have classically been described as the causative agents, and obligate anaerobic bacteria have also been included [10]. Lip swellings are commonly diagnosed as angioedema; Bruno et al. reported a case of lung pneumonia as the consequence of a lower lip abscess which was mistakenly diagnosed as angioedema [11]. Also, Lee et al. reported a case of facial NF as the consequence of a facial wound over the upper lip, but not involving the lip itself [12].We report a unique case of lower lip cellulitis, which was initially misdiagnosed as angioedema and subsequently progressed to lower lip necrotizing fasciitis.

## Case presentation

A 19-year-old black female presented with painful lower lip swelling of 2 weeks duration. She was initially seen by a physician whom she claimed had given her anti-allergic medication and painkillers in the form of tablets for 1 week, but the situation worsened and the swelling and pain increased. She was then prescribed intravenous penicillin for another week with no improvement, after which she presented to our hospital for assessment and further management. There was lower lip swelling with discharge from the exfoliated crusted lower lip surface. The condition had started as a small pimple on her chin just below the lower lip on the left side which was painful and increasing in size. She attempted to open it and did not use any antibiotic. After 48 h she became febrile and her lower lip became swollen. She went first to a physician, then presented to our dental hospital. The patient’s medical and family history was non-contributory, and there was no history of trauma, hospitalization or current medication except those prescribed by the physician. She was lactating and had a 6 month old boy.

**Fig. 1:**
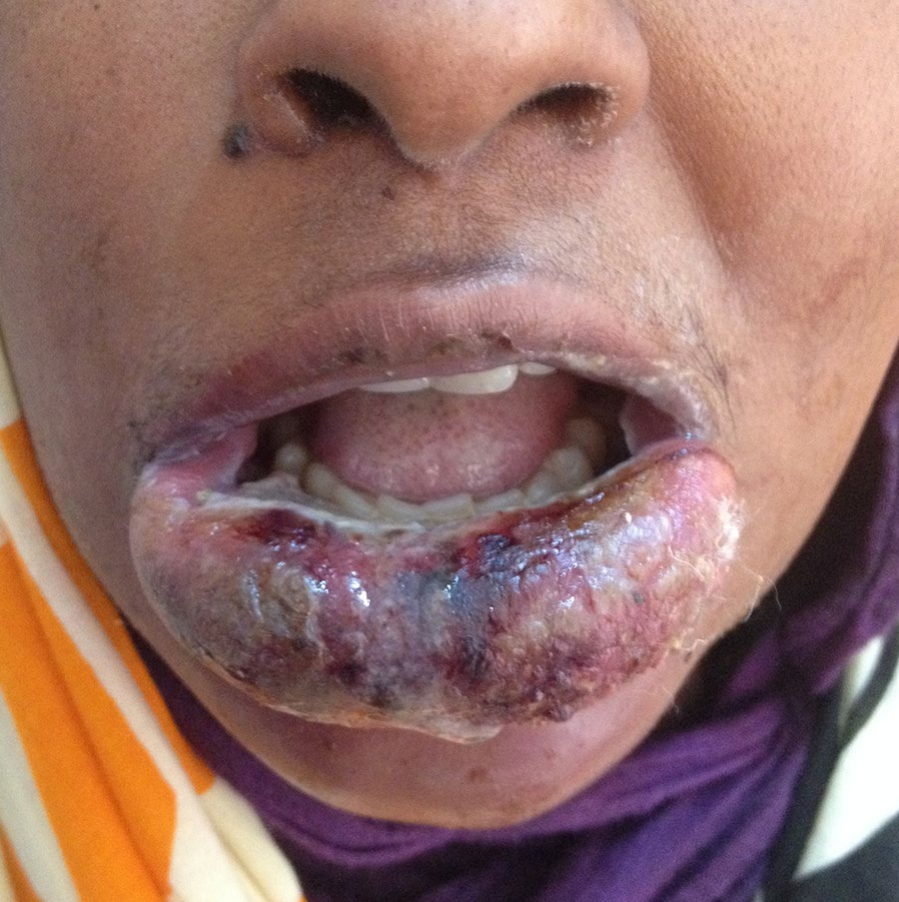
Patient with painful lower lip swelling

**Fig. 2:**
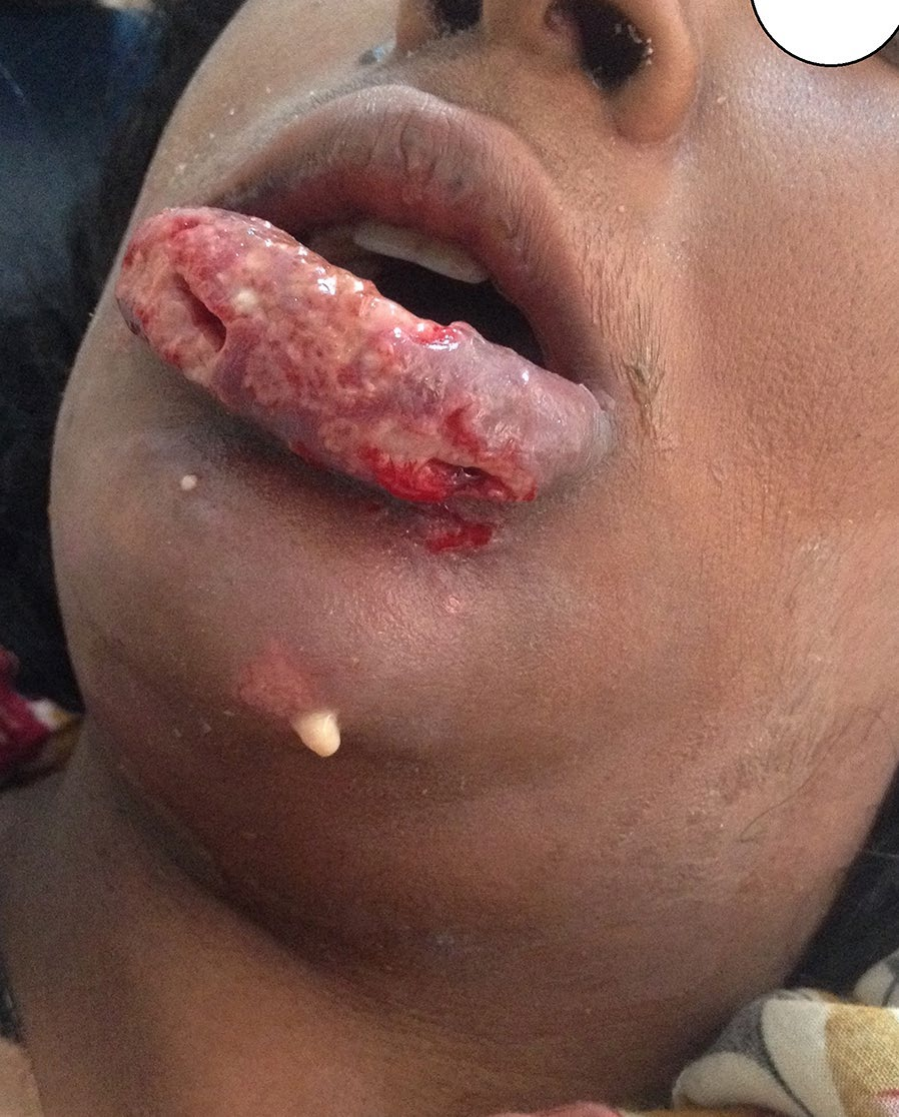
Sinus discharging pus at the *left side* of chin

The patient’s oral cavity was examined carefully and good oral hygiene was noted. There were no obvious odontogenic causes or lip paraesthesia. Panoramic radiograph was done to exclude odontogenic causes and was insignificant. A thorough facial examination followed and a small crusted pimple was noticed on the left side of the chin. There was extensive swelling involving the whole lower lip and extending to involve the chin and submental area (Fig. 1) with pus discharging from the left side of the chin just beneath the site of the pimple (Fig. 2). The patient’s blood investigations were negative for HIV and hepatitis, with a TWBC of (25.8×), random blood glucose of 190 mg/dl and a creatinine of 0.7 mg/dl. She was anemic (10 g/dl), mildly hyponatremic (134 mmol/l) and hypokalemic (2.5 mmol/l). Potassium correction was started within travenous potassium chloride at 10 meq/l over 4 h for 1 day, after which she was shifted to diet supplements. Normal saline 500 ml/day was given for 2 days, as well as ferrous sulphate tablets. Electrolyte and blood levels were back to normal after 6 days (K = 3.5 mmol/l, Na = 137 mmol/l and Hb = 12 g/dl.

**Fig. 3:**
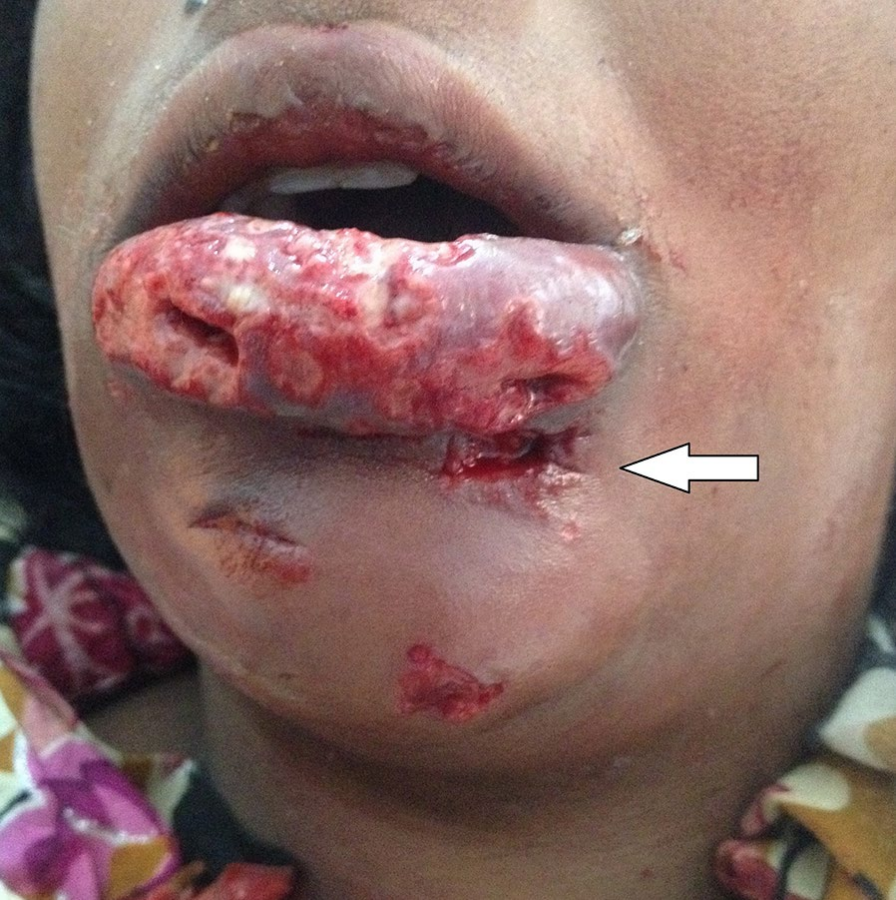
Surgical debridement and multiple skin incision

**Fig. 4:**
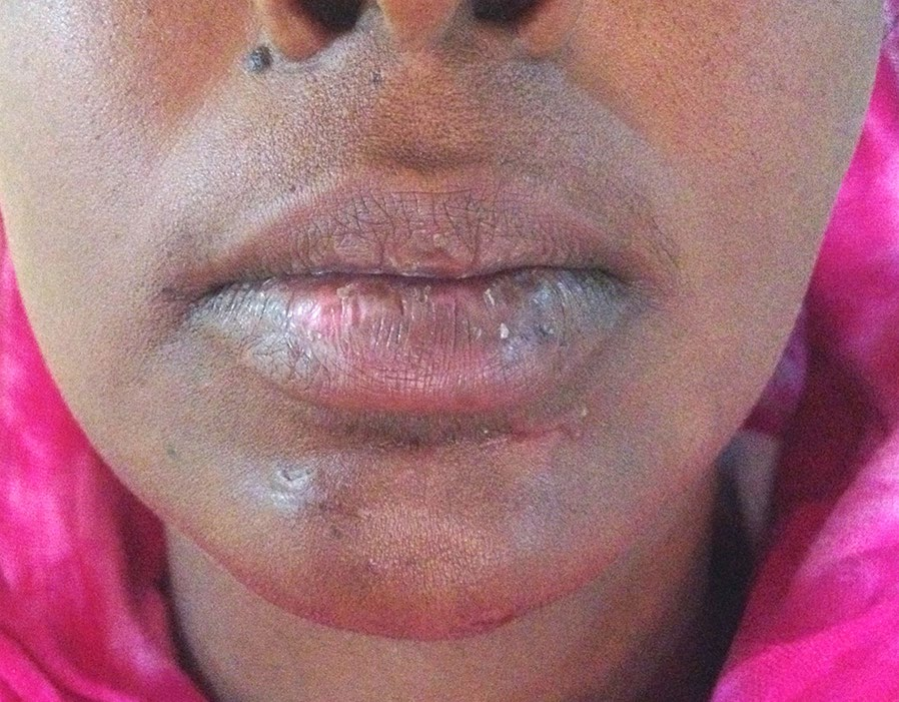
Patient appearance 4 months postoperatively

Based on the history and clinical presentation, the diagnosis of necrotizing fasciitis was made and a swab of the site was taken for culture and sensitivity. Treatment started with lower lip debridement and exploration where necrosis of the middle part of the lower lip was noted. A segment of necrotic fascia on the left side of the chin was found and removed with preservation of the overlying skin except over the acne-affected area where skin was removed (Fig. 3). Multiple mental and submental incisions were made for drainage. Irrigation with normal saline was done and corrugated rubber drains were inserted and secured. The patient was admitted to the hospital where intravenous fluids and an empirical triple antibiotic regimen were started immediately (Ceftazidime 1Gm/day, Metronidazole 500 mg 8 hourly and Gentamycin 80 mg/day), and continued after culture and sensitivity results revealed no microbial growth. After 3 days, further tissue necrosis was noticed, so a second debridement was done with consequent tissue loss over the middle upper surface of the lower lip. Additionally, irrigation with hydrogen peroxide followed by normal saline wash was performed. Ten days later, dramatic improvement was seen, where only the Gentamycin was stopped and irrigation was continued with normal saline and topical application of Tetracycline ointment for 7 days. The drain was removed on day 19 and the patient was discharged on the 20th day on oral antibiotics for another week. On follow up, the wound showed good healing by secondary intention and there was no need for reconstruction (Fig. 4).

## Discussion

Necrotizing fasciitis is an infection of the deeper tissues that results in progressive destruction of muscle fasciae and overlying subcutaneous fat. The muscle tissue itself is frequently spared because of its generous blood supply [13].

A definitive diagnosis of NF is established surgically with visualization of fascial planes and subcutaneous tissue. However, clinical clues and diagnostic tools should be used in combination to help make an early diagnosis [1]. The clinician must be highly suspicious regarding any patient presenting with a rapidly spreading swelling, erythema and fever with palpation of the wound to check for crepitus which may indicate subcutaneous gas production. The LRINEC score (Laboratory Risk Indicator for Necrotizing Fasciitis) was developed by Wong et al. to detect early cases of NF clinically. The variables used are routinely measured to assess severe soft tissue infections. Patients with a LRINEC score of >6 should be carefully evaluated for the presence of necrotizing fasciitis [14]. A LRINEC score of 7 was detected in our patient (excluding C reactive protein which was not done due to financial issues) which was suggestive of NF before surgical exploration was started.

Treatment of NF consists of early and aggressive debridement of necrotic tissue, together with broad-spectrum antibiotics and fluids support. It is important to mention that NF can progress in an insidious manner and by the time it has been diagnosed the condition would have progressed to a late stage. In this patient’s case, immediate surgical debridement of the whole necrotic tissue deep to the orbiculariousor is muscle was done until healthy tissue was visible. Extensive fasciotomy with exposure and exploration of all involved compartments was done. Absence of growth in culture and sensitivity results could be explained by the effect of the antibiotics prescribed before she presented to the hospital. Daily dressing was performed and debridement was kept to the minimum essential to minimize the lip defect that might result from generous tissue removal.

Patients with NF usually have an associated systemic condition such as diabetes mellitus, arteriosclerosis, obesity, metastatic neoplasm, old age, hypothyroidism, alcoholism, neoplasms, cirrhosis, drug abuse, poor nutritional state, or use of corticosteroids [8]. In our case the patient was only mildly anemic and malnourished; factors that most likely predisposed to necrotizing fasciitis. Therefore, other therapeutic considerations were included to manage the hyponatremia and hypokalemia. Multivitamins were prescribed in order to improve the patient’s general health. Anemia was treated by oral supplements. Hemolysis of erythrocytes may occur as a result of the actions of bacterial enzymes, resulting in anemia that may require transfusion.

Facial NF is generally due to a dental or pharyngeal abscess (surgical or post-traumatic), radiotherapy, or an unknown cause [8]. Lower lip NF is rare, but lip cellulitis as a result of chin acne infection is common especially in young adults. In the present case, it was initially misdiagnosed as angioedema. Such diagnosis resulted in the inappropriate management and delayed the proper treatment.

## Conclusion

This case illustrates a consequence of misdiagnosis of NF. When confronted with lip swelling the clinician must consider the broad spectrum of differential diagnoses and consider a developing NF on the list. Triple antibiotic therapy, investigations, correction of deficiencies, dressing and debridement are essential components of the proper management of NF.

## Abbreviations

NF: necrotizing fasciitis
HIV: human immunodeficiency virus
TWBC: total white blood cell
K: potassium
Na: sodium
Hb: hemoglobin
LRINEC: Laboratory risk indicator for necrotizing fasciitis
